# Establishment and validation of a predictive nomogram for polyuria during general anesthesia in thoracic surgery

**DOI:** 10.1186/s13019-024-02833-5

**Published:** 2024-07-03

**Authors:** Jiajie Li, Zongwang Zhang

**Affiliations:** 1 Department of Anesthesiology, Xinxiang Central Hospital, Xinxiang, Henan Province 453000 China; 2 Department of Anesthesiology, Liaocheng people’s Hospital Affiliated to Shandong First Medical University, No. 67, Dongchang West Road, Dongchangfu District, Liaocheng, Shandong Province 252004 China

**Keywords:** Polyuria predictive model, Nomogram, Receiver operating characteristic curve, Hosmer–Lemeshow test, Decision curve analysis

## Abstract

**Background:**

To develop and evaluate a predictive nomogram for polyuria during general anesthesia in thoracic surgery.

**Methods:**

A retrospective study was designed and performed. The whole dataset was used to develop the predictive nomogram and used a stepwise algorithm to screen variables. The stepwise algorithm was based on Akaike’s information criterion (AIC). Multivariable logistic regression analysis was used to develop the nomogram. The receiver operating characteristic (ROC) curve was used to evaluate the model’s discrimination ability. The Hosmer–Lemeshow (HL) test was performed to check if the model was well calibrated. Decision curve analysis (DCA) was performed to measure the nomogram’s clinical usefulness and net benefits. *P* < 0.05 was considered to indicate statistical significance.

**Results:**

The sample included 529 subjects who had undergone thoracic surgery. Fentanyl use, gender, the difference between mean arterial pressure at admission and before the operation, operation type, total amount of fluids and blood products transfused, blood loss, vasopressor, and cisatracurium use were identified as predictors and incorporated into the nomogram. The nomogram showed good discrimination ability on the receiver operating characteristic curve (0.6937) and is well calibrated using the Hosmer–Lemeshow test. Decision curve analysis demonstrated that the nomogram was clinically useful.

**Conclusions:**

Individualized and precise prediction of intraoperative polyuria allows for better anesthesia management and early prevention optimization.

## Background

As intraoperative hypotension is associated with increased morbidity and mortality, perioperative monitoring and regulation of circulatory function is an important task for the anesthesiologist [1]. As patients are routinely instructed to abstain from eating and drinking before surgery, intraoperative polyuria is a rare perioperative complication that can cause negative volume balance in patients. It can be secondary to drug therapy or surgery and may cause significant hypovolemia and lead to patient distress, intraoperative hypotension, electrolyte disturbances (particularly hypernatremia), circulatory collapse, and even death [2]. Anesthesiologists must use large amounts of vasoactive drugs or infuse large volumes of fluids intraoperatively to maintain the perfusion of patients’ vital organs.

In adults, intraoperative polyuria is typically defined as urine volume ≥ 2.5 mL/kg/h according to a study [3] or as ≥ 3 L/24 h or ≥ 40–50 mL/kg/24 h according to several other studies [4–6]. Under intraoperative anesthesia, it is challenging for the anesthesiologist to detect the patient’s symptoms. Many intermediate and major surgeries have a long operative time with a long infusion time of anesthetic drugs and large accumulation doses; this may lead to massive loss of water and electrolytes in the body and increased plasma osmolality, consequently resulting in severe neurological symptoms, such as weakness, lethargy, myalgias, and coma^2^. Acute increases in serum sodium levels can cause cerebral dehydration, potentially resulting into rupture of dural bridging veins, cerebral hemorrhages, venous sinus thrombosis, cerebral demyelination, and rhabdomyolysis [7]. There have been case reports wherein the use of certain anesthetic drugs was suspected to cause intraoperative polyuria, mainly including dexmedetomidine [8–15], ketamine [16–19], propofol [20–23], sevoflurane [24, 25], remifentanil [26], and fentanyl [27]. A review paper published in the journal Anesthesia and Analgesia in 2021 reviewed 24 case reports of intraoperative polyuria due to anesthetic drugs and their possible mechanisms [2]. However, as the factors associated with intraoperative polyuria during general anesthesia remain unclear, we wanted to identify the factors influencing intraoperative polyuria, develop a predictive model, and validate it in this retrospective study.

## Methods

### Patient selection and study design

This retrospective study was aimed at establishing and validating a predictive nomogram for polyuria. It was conducted in Liaocheng People’s Hospital and registered with the Chinese Clinical Trial Registry (No. ChiCTR2100053977). The study sample comprised patients who underwent thoracic surgery in Liaocheng People’s Hospital between January 2020 and December 2020. This study was approved by Liaocheng People’s Hospital Ethics Committee (Approval No. 2,021,185). All methods were performed in accordance with the relevant guidelines and regulations. All participants signed the informed consent.

Inclusion criteria were as follows: patients with urine volume records on the anesthesia sheet and patients aged over 18 years scheduled to undergo thoracic surgery.

**Fig. 1:**
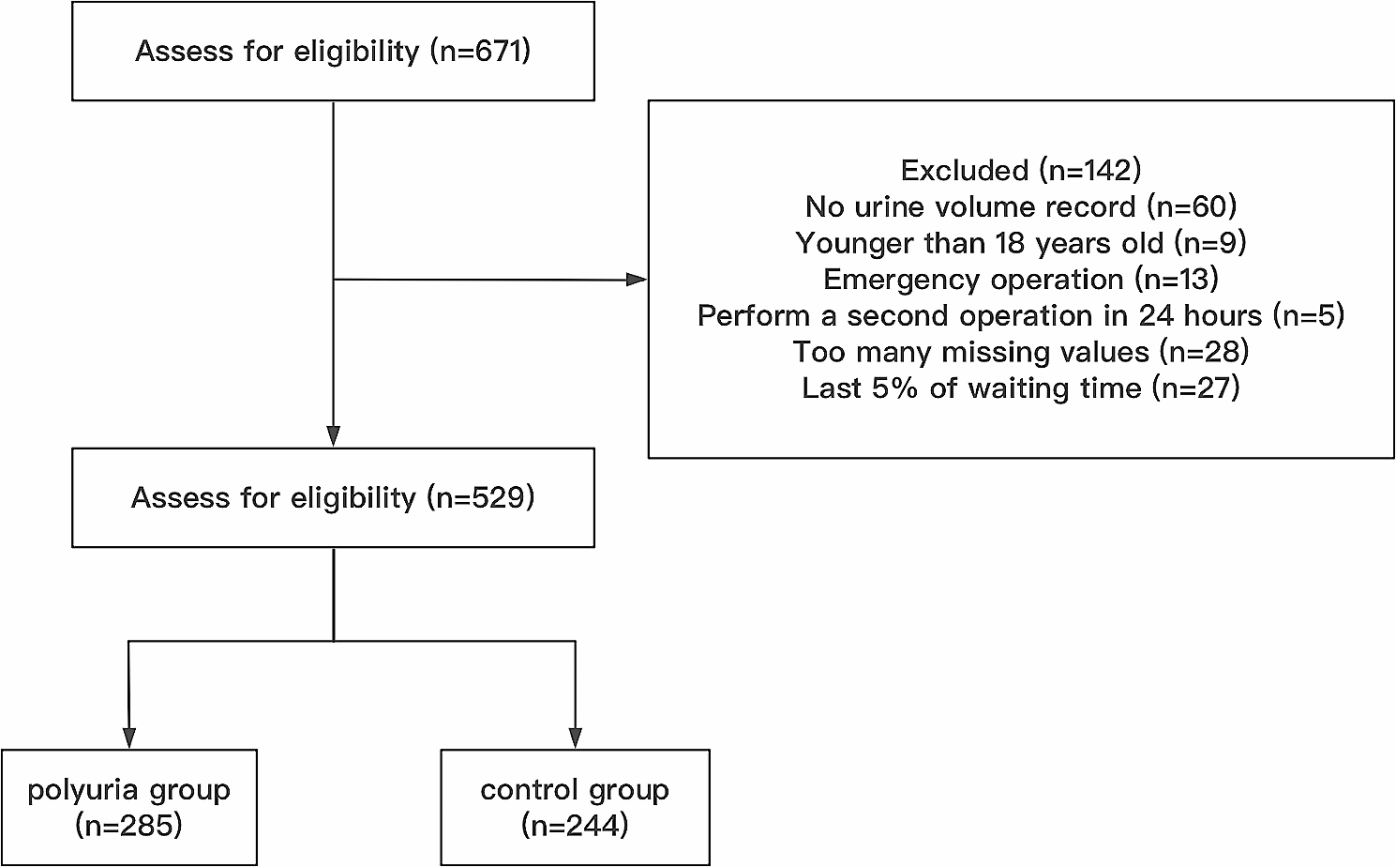
Number of participants enrolled and divided into groups

We excluded patients who underwent emergency operation, for whom re-operation was performed within 24 h. We excluded patients with preoperative comorbid end-stage renal disease (i.e., eGFR < 15 mL/min/1.73 m^2^ or on dialysis). Patients with preoperative central nervous system diseases (e.g., pituitary tumors) were also excluded. In addition, due to the different anesthesia record sheet habits of each doctor and differences in medication habits, some drugs were only used in very few patients and therefore placed in the entire data set such drugs have too many missing values and this sample will also be excluded. Furthermore, as the waiting time for some patients was long enough to influence the outcomes, we excluded 5% if of the patients with the longest waiting time (Fig. 1).

In this study, we defined polyuria as urine volume ≥ 2.5 mL/kg/h^3^ in our study. According to the urine volume, 285/529 patients were assigned to the polyuria group and 244/529 patients to the control group.

### Study predictors

We recorded several candidate predictors, including demographic characteristics (gender, age, hypertension, diabetes, coronary heart disease, smoking history, and alcohol intake), preoperative biochemical examination (serum creatinine level, hemoglobin level, and platelet count), preoperative patient anxiety factors (waiting time, the difference between MAP at admission and before the operation, and the difference between heart rate at admission and before the operation), surgery-related factors [operation type (endoscopy surgery or open surgery) and surgical position (supine position or prone position)], anesthesia-related factors (fasting time, MAP after anesthesia induction, and heart rate after anesthesia induction), volume factors (total amount of fluids and blood products transfused and blood loss), and the anesthesia drug used [dexmedetomidine, vasopressor (deoxyepinephrine and norepinephrine), fentanyl, cisatracurium, and propofol use].

### Statistical analysis

Our sample size was calculated ensuring at least 10 outcome events per variable, and with there being 27 variables, we required more than 270 cases as per the events per variable principle. With our sample size of 529 cases, this requirement was easily met and could yield robust estimates. Collected data were collated in an Excel sheet (Microsoft, Redmond, WA, USA). Statistical analyses were performed using R version 4.2.1 and Stata version 17.0. Variables like gender, hypertension, diabetes, coronary heart disease, smoking history, alcohol intake, operation type, surgical position, dexmedetomidine, and vasopressor were categorical variables. In addition, variables including waiting time, age, serum creatinine level, hemoglobin level, platelet count, the difference between MAP at admission and before the operation, the difference between heart rate at admission and before the operation, fasting time, total amount of fluids and blood products transfused, blood loss, fentanyl use, cisatracurium use, propofol use, MAP after anesthesia induction, and heart rate after anesthesia induction were continuous variables.

All variables were selected based on our clinical work and case reports searched from PubMed. Wilcoxon rank sum test was used for continuous variables. Pearson’s Chi-squared test was used for categorical variables. After selecting potential risk factors, we performed a univariate analysis to select the variables. A statistical significance level of 0.20 was used to select variables for the model [28], and multivariable regression analysis was used to identify variables with a statistical significance level of < 0.05 and incorporate them into the nomogram. The whole dataset was used to develop the predictive nomogram and used a stepwise algorithm to filter variables. Although cisatracurium use was only borderline insignificant (*P* = 0.065), we incorporated it into the nomogram according to the Akaike’s information criterion (AIC). The AIC value for the final nomogram was minimized with the fewest number of variables. A nomogram was constructed using the “rms” R package. The receiver operating characteristic (ROC) curve was used to evaluate the model’s discrimination. The HL test was used to verify the calibration of the model. Decision curve analysis (DCA) was performed to assess the nomogram’s clinical usefulness and net benefits.

## Results

### Clinical characteristics of patients

Among the 671 patients screened, 529 patients met the inclusion criteria, and 285/529 patients were assigned to the polyuria group and 244/529 to the control group. Variables with *P* < 0.2 were screened using univariate analysis and considered statistically significant (Table 1). Variables were screened for multifactorial regression analysis in conjunction with clinical reality. *P* < 0.05 was considered to indicate statistical significance (Table 2). These data were used to develop the nomogram predictive model. Fentanyl [log(OR) = 0.38, β = 1.46, 95% CI (0.22, 0.54), *P* < 0.001] was identified as the most significant risk factor for intraoperative polyuria, and with each unit increase of fentanyl dosage administered, the probability of intraoperative polyuria was found to increase by 46%.

**Table 1:** Results of univariate analysis

Characteristics	Overall, *N* = 529^*1*^	0, *N* = 244^*1*^	1, *N* = 285^*1*^	*P* value^2^
Urine (mL)	2.60 (1.70, 4.00)	1.70 (1.30, 2.10)	3.90 (3.10, 5.20)	< 0.001
Waiting time (days)	5 (3, 7)	5 (3, 7)	5 (3, 7)	0.638
Gender				< 0.001
0 (Female)	222 (42%)	78 (32%)	144 (51%)	
1 (Male)	307 (58%)	166 (68%)	141 (49%)	
Age	62 (55, 68)	63 (55, 68)	62 (55, 69)	0.521
HBP				0.834
0 (No)	358 (68%)	164 (67%)	194 (68%)	
1 (Yes)	171 (32%)	80 (33%)	91 (32%)	
CHD				0.563
0 (No)	479 (91%)	219 (90%)	260 (91%)	
1 (Yes)	50 (9.5%)	25 (10%)	25 (8.8%)	
DM				0.241
0 (No)	481 (91%)	218 (89%)	263 (92%)	
1 (Yes)	48 (9.1%)	26 (11%)	22 (7.7%)	
Cr (µmol/L)	62 (53, 70)	63 (54, 72)	60 (53, 69)	0.041
PLT (×10^9^/L)	232 (196, 281)	223 (196, 276)	237 (197, 283)	0.279
Hb (g/L)	133 (125, 144)	136 (126, 147)	131 (124, 140)	< 0.001
Smoking history				0.085
0 (No)	318 (60%)	137 (56%)	181 (64%)	
1 (Yes)	211 (40%)	107 (44%)	104 (36%)	
Alcohol intake				0.095
0 (No)	328 (62%)	142 (58%)	186 (65%)	
1 (Yes)	201 (38%)	102 (42%)	99 (35%)	
MAP^*^ (mmHg)	5 (-3, 13)	3 (-6, 13)	6 (-1, 14)	0.005
HR^*^ (bpm)	2 (-5, 13)	2 (-5, 13)	3 (-5, 11)	0.942
Fasting time (h)	8 (8, 12)	8 (8, 12)	8 (8, 12)	0.731
Operation type				0.021
0 (Endoscopic surgery)	99 (19%)	56 (23%)	43 (15%)	
1 (Open surgery)	430 (81%)	188 (77%)	242 (85%)	
Surgical position				0.382
1 (Supine position)	52 (9.8%)	21 (8.6%)	31 (11%)	
2 (Prone position)	477 (90%)	223 (91%)	254 (89%)	
Total amount of fluids and blood products transfused (mL)	1,500 (1,000, 2,000)	1,500 (1,000, 2,000)	1,500 (1,000, 2,000)	0.548
Blood loss (mL)	100 (50, 100)	100 (50, 150)	50 (50, 100)	0.030
Dexmedetomidine				0.636
0 (No use)	69 (13%)	30 (12%)	39 (14%)	
1 (Use)	460 (87%)	214 (88%)	246 (86%)	
Vasopressor				0.248
0 (No use)	284 (54%)	121 (50%)	163 (57%)	
1 (Use deoxyepinephrine)	161 (30%)	77 (32%)	84 (29%)	
2 (Use norepinephrine)	73 (14%)	40 (16%)	33 (12%)	
3 (Use both)	11 (2.1%)	6 (2.5%)	5 (1.8%)	
Fentanyl use (µg/kg)	4.55 (3.64, 5.52)	4.29 (3.37, 5.15)	4.76 (3.92, 5.83)	< 0.001
Cisatracurium use (mg/kg)	0.32 (0.25, 0.40)	0.31 (0.25, 0.40)	0.32 (0.25, 0.42)	0.406
Propofol use (mg/kg)	1.65 (1.00, 2.07)	1.60 (0.97, 2.00)	1.74 (1.03, 2.17)	0.029
MAP after anesthesia induction (mmHg)	89 (80, 98)	89 (79, 98)	89 (80, 99)	0.456
HR after anesthesia induction (bpm)	73 (64, 84)	73 (63, 86)	73 (64, 82)	0.522

^*1*^ Median (IQR); n (%)

^*2*^ Wilcoxon rank sum test; Pearson’s Chi-squared test

^*^ the difference between MAP or HR at admission and before the operation

Continuous variables were expressed as median (IQR). Categorical variables were expressed as n (%)

Abbreviations: HBP, hypertension; CHD, coronary heart disease; DM, diabetes mellitus; Cr, creatinine; PLT, platelet; Hb, hemoglobin; MAP, mean arterial pressure; HR, heart rate; IQR, interquartile range

Wilcoxon rank sum test was used for continuous variables. Pearson’s Chi-squared test was used for categorical variables. *P* < 0.2 indicated the statistical significance of the variable

**Table 2:** results of multifactorial analysis

Characteristics	log(OR)^1^	95% CI^1^	*P* value
Fentanyl use (µg/kg)	0.38	0.22, 0.54	< 0.001
Waiting time (days)	0.00	-0.07, 0.08	> 0.9
Gender			
0 (Female)	—	—	
1 (Male)	-0.86	-1.5, -0.24	0.007
Age	-0.01	-0.03, 0.02	0.6
HBP	0.06	-0.37, 0.49	0.8
CHD	-0.24	-0.91, 0.44	0.5
DM	-0.36	-1.0, 0.31	0.3
Cr (µmol/L)	0.01	-0.01, 0.03	0.4
PLT (×10^9^/L)	0.00	0.00, 0.00	0.4
Hb (g/L)	-0.01	-0.02, 0.01	0.3
Smoking history	0.34	-0.27, 1.0	0.3
Alcohol intake	0.05	-0.53, 0.62	0.9
MAP^*^ (mmHg)	0.02	0.00, 0.03	0.028
HR^*^ (bpm)	0.00	-0.02, 0.01	0.8
Fasting time (h)	0.00	-0.07, 0.08	0.9
Operative style	0.50	-0.02, 1.0	0.062
Surgical position	-0.26	-0.93, 0.38	0.4
Total amount of fluids and blood products transfused (mL)	0.00	0.00, 0.00	0.034
Blood loss (mL)	0.00	0.00, 0.00	0.040
Dexmedetomidine	-0.01	-0.58, 0.55	> 0.9
Vasopressor	-0.26	-0.51, -0.01	0.045
Cisatracurium use (mg/kg)	-2.0	-4.1, 0.11	0.065
Propofol use (mg/kg)	0.07	-0.13, 0.28	0.5
MAP after anesthesia induction	0.00	-0.02, 0.02	> 0.9
HR after anesthesia induction	-0.01	-0.02, 0.01	0.4

^*1*^ OR = odds ratio, CI = confidence interval

^*^ the difference between MAP or HR at admission and before the operation

Abbreviations: HBP, hypertension; CHD, coronary heart disease; DM, diabetes mellitus; Cr, creatinine; PLT, platelet; Hb, hemoglobin; MAP, mean arterial pressure; HR, heart rate; OR, odds ratio; CI, confidence interval

*P* < 0.05 considered the variable statistically significant

### Building predictive models

**Fig. 2:**
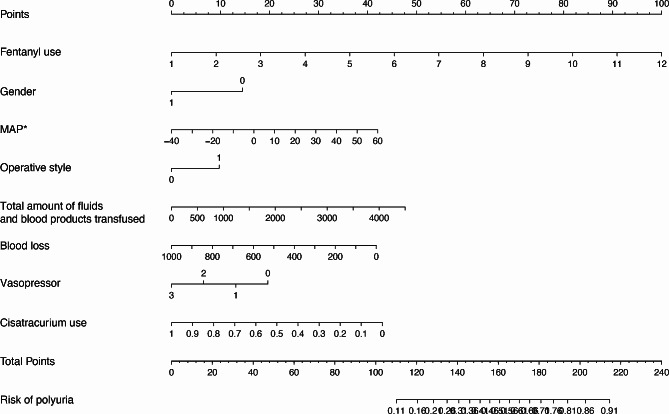
The nomogram estimates the probability of intraoperative polyuria due to each risk factor. We identified the predictor points on the bottom point scale that correspond to each patient variable and added them. The % corresponding to the total score represents the probability of intraoperative polyuria occurring in that patient

The variables filtered out using the stepwise algorithm and AIC were fentanyl use, gender, the difference between MAP at admission and before the operation, operation type, total amount of fluids and blood products transfused, blood loss, vasopressor, and cisatracurium use. These have been represented using a nomogram. The predicted probability of developing intraoperative polyuria ranged from 11 to 91% (Fig. 2).

### Differentiation efficiency of the model

**Fig. 3:**
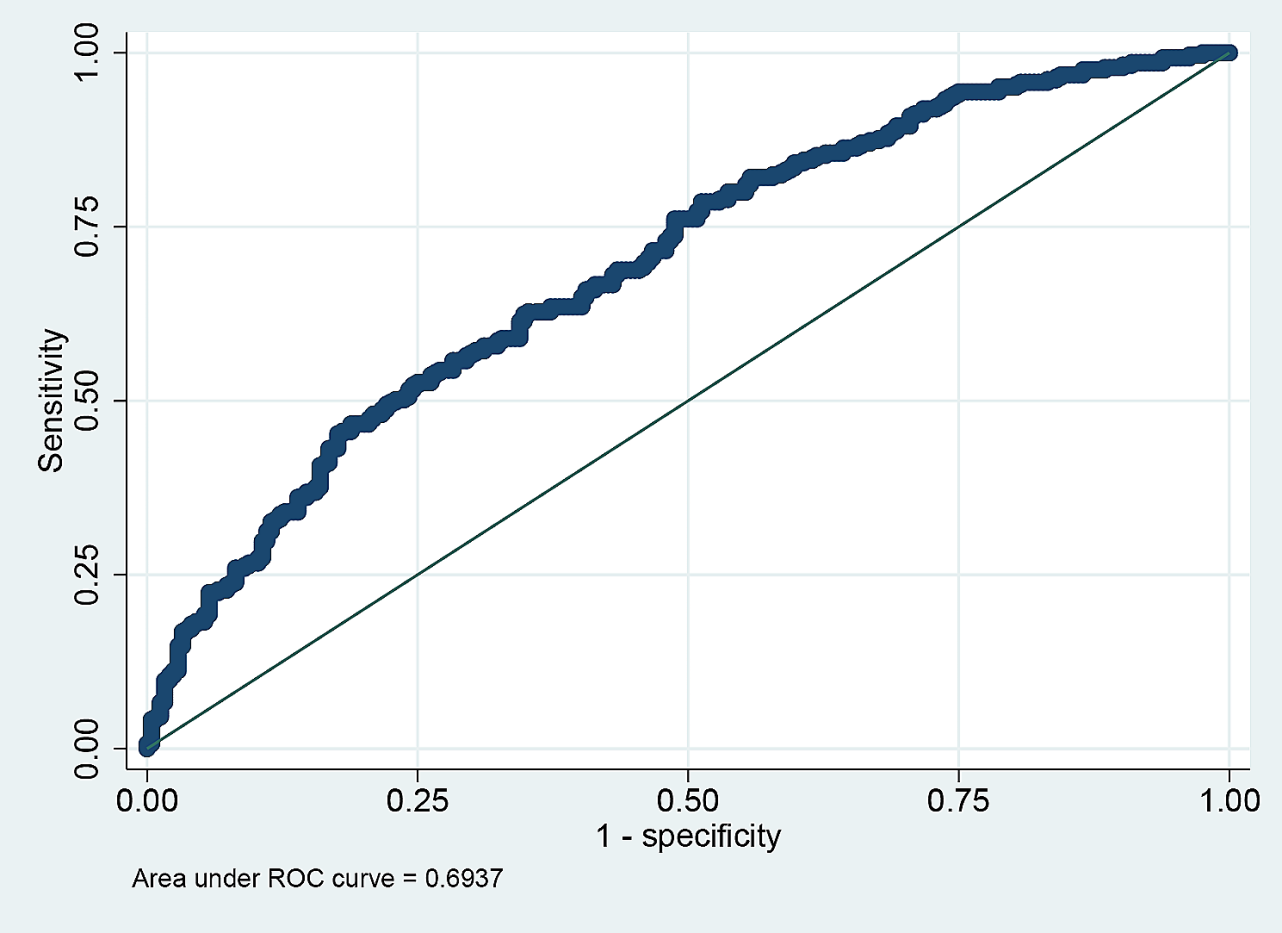
The nomogram had good discriminative power with the area under the receiver operating characteristic curve of 0.6937

The ROC curve is plotted in Fig. 3. The nomogram had good discriminative power with the area an under the ROC curve of 0.69.

### Calibration efficiency of the model

**Fig. 4:**
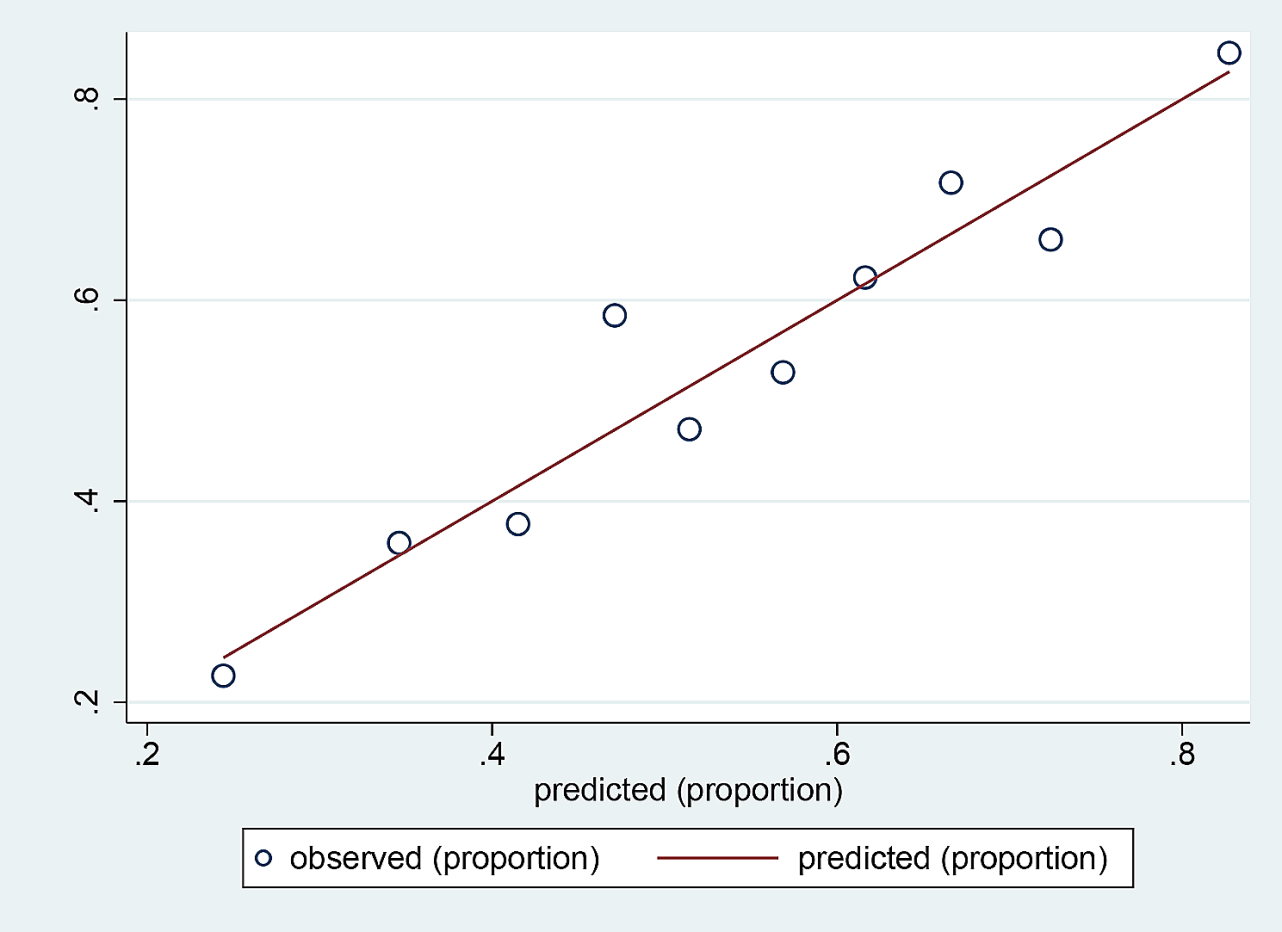
The HL curve was used to validate the calibration of the nomogram. The X-axis represents the predicted values. The Y-axis represents observed values. In a well-calibrated nomogram, the scatter points should be arranged along the red 45° line

**Fig. 5:**
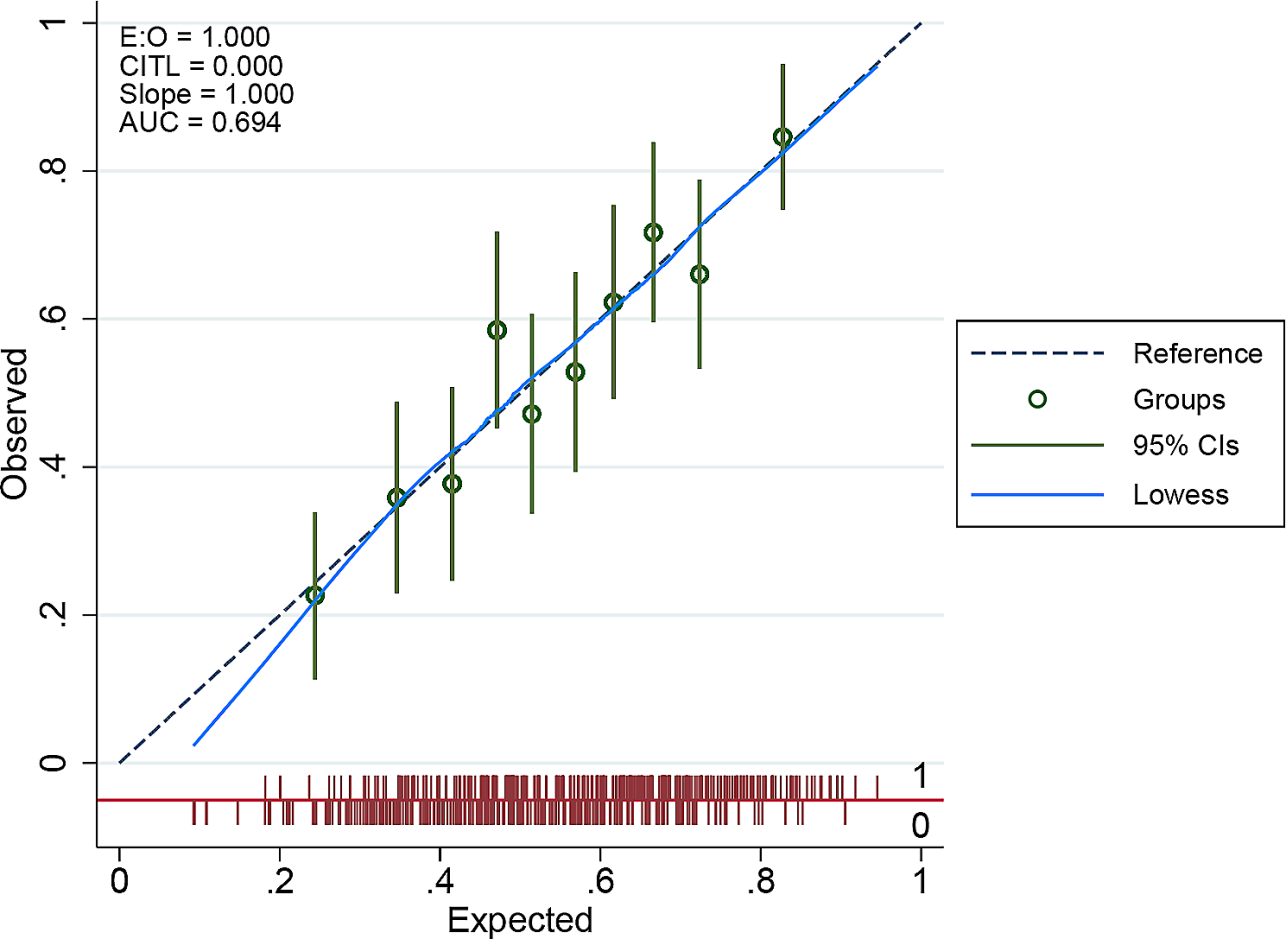
The plot shows a well-calibrated nomogram. The X-axis indicates the expected value, and the Y-axis indicates the observed value. The green circles represent the 10 groups. The green vertical line represents the 95% CI of the group

The HL curve is shown in Fig. 4. The plot shows the relationship between predicted and observed values. The line drawn along 45° indicates perfect calibration of the model. The 10 sets of data are distributed above and below the line. In Fig. 5, the 10 green circles represent the 10 groups. The green vertical line represents the 95% CI of these groups. The blue dashed lines represent the ideal model reference values above and below which the 10 groups are distributed. This light blue line represents a smooth-fitting line comprising 10 points. The red vertical line at the bottom represents the distribution of all samples, with 1 indicating the polyuria group sample and 0 indicating the control group sample. These two plots indicate that this model is well calibrated.

### Clinical decision analysis capabilities of the model

**Fig. 6:**
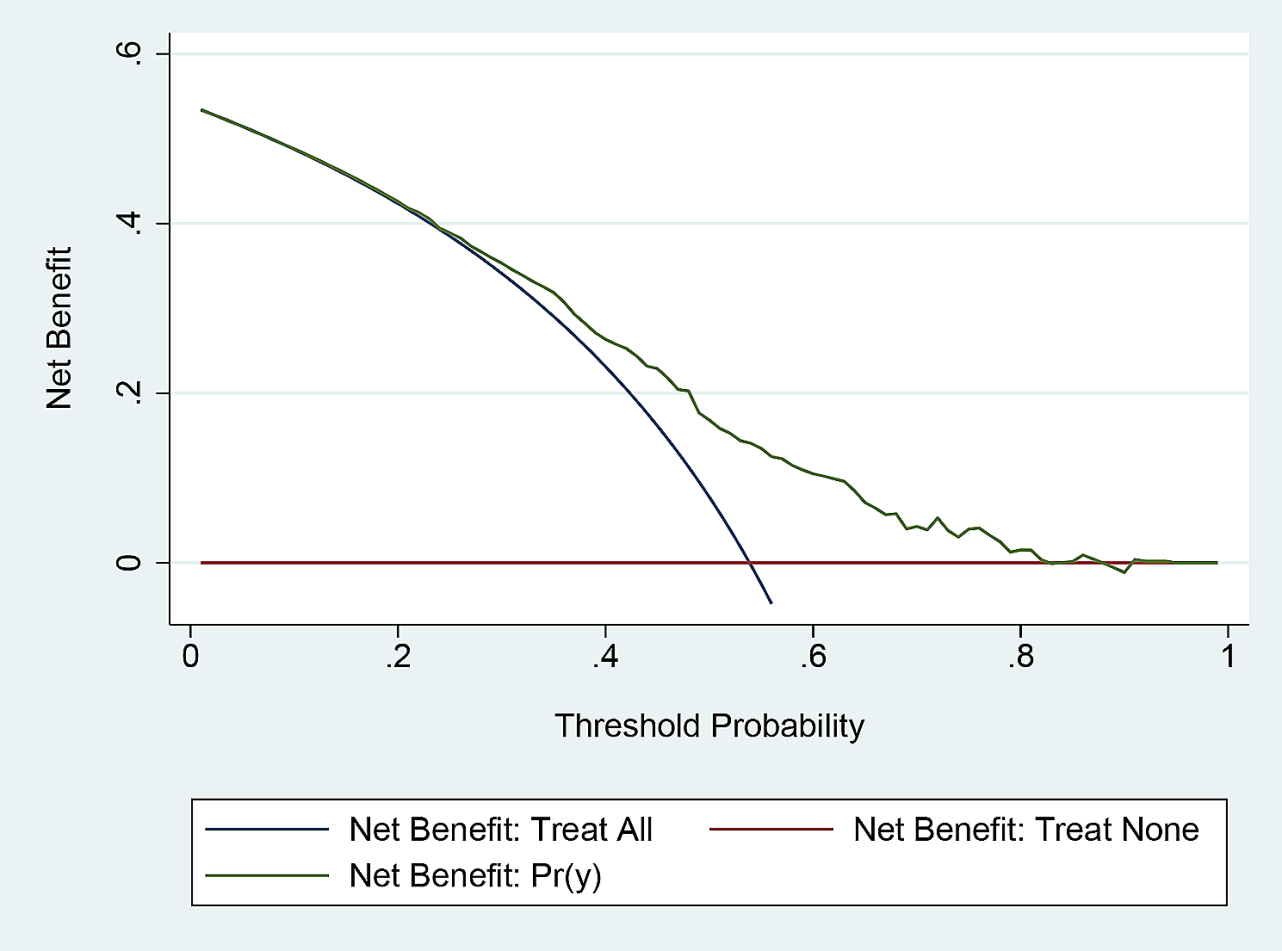
Decision curve analysis for the nomogram. The Y-axis represents the net benefit. The green line represents the nomogram. The blue line represents the assumption that all patients have polyuria. The red line represents the assumption that no patients have polyuria

The DCA for the nomogram is presented in Fig. 6. We performed DCA on our prediction model to assess the net benefit to the patient. The X-axis represents threshold probability, and the Y-axis represents the net benefit. The findings reveal that the nomogram model has obvious net benefit for almost all threshold probabilities, particularly in threshold probabilities of 20–80%.

## Discussion

expandIn this study, we established and validated a predictive nomogram for polyuria during general anesthesia in thoracic surgery. It shows good discrimination ability and is well calibrated. This will provide useful guidance for subsequent anesthetic management and prevention of intraoperative polyuria and provide additional benefits for perioperative volume management of patients.

expandThe results revealed fentanyl use, gender, the difference between MAP at admission and before the operation, operation type, total amount of fluids and blood products transfused, blood loss, vasopressor, and cisatracurium use as the predictors for polyuria in thoracic surgery. This trial revealed fentanyl use as the most important risk factor for intraoperative polyuria. Therefore, we searched for mechanisms relating fentanyl to intraoperative urine volume.

expandExisting literature on the association of fentanyl with intraoperative urine volume suggests that the underlying mechanism is associated with arginine vasopressin (AVP) release. Lehtinen et al. [27] suggested that inducing small-dose fentanyl anesthesia leads to AVP release. This results in concentrated urine and reduced urine volume, which can be antagonized by preoperative naloxone use [27]. Lange et al. [29] found a significant increase in plasma antidiuretic hormone (ADH) levels during coronary artery bypass surgery in patients given fentanyl. High doses of alfentanil and sufentanil allowed stable cardiovascular kinetics during anesthesia, throughout the procedure (including extracorporeal circulation), and at the end of the procedure, blocking the increase in ADH and GH levels [29]. Stanley et al. [30] reported that plasma ADH levels during coronary surgery did not significantly differ from pre-anesthesia levels for any fentanyl dose. The authors concluded that high-dose fentanyl anesthesia blocked the increase in plasma ADH levels [30]. Fentanyl had no significant effect on plasma ADH levels [30]. Kono et al. [31] found that in patients undergoing coronary surgery, urinary osmolality and urinary sodium concentration decreased and urine volume increased after fentanyl infusion in the second operative period; however, no significant change in AVP levels was identified. The increased urine volume and decreased urinary sodium excretion during fentanyl anesthesia also indicated hydronephrosis, which was reflected by decreased urinary osmolality [31]. They concluded that fentanyl can block the stress response to surgery and that high-dose fentanyl had no significant effect on the release of AVP [31]. Furthermore, they showed that patients in the fentanyl group had the lowest urinary sodium concentration and significantly higher urine volume than patients in other groups [31]. Conclusions on the associations of fentanyl with AVP levels and intraoperative urine volume differ among various studies. Therefore, the exact mechanism warrants further investigation. Due to the high trauma of thoracic surgery, when analgesia is inadequate, additional fentanyl dosage is typically used to reduce the stress. This retrospective study showed that the additional fentanyl dosage increased the intraoperative urine output in patients. Ultrasound-guided nerve blocks have become widely available in recent years and can effectively reduce intraoperative and postoperative pain levels in patients undergoing general anesthesia for thoracic surgery, resulting in less fentanyl use. The present study suggests that adequate preoperative nerve blocks can reduce the dosage of intraoperative analgesic fentanyl and further reduce the occurrence of intraoperative polyuria.

expandNext, upon exploring the occurrence of intraoperative polyuria in both sexes separately, female patients were found to have a greater propensity to have intraoperative polyuria than male patients. The vast majority of patients undergoing thoracic surgery have lung or esophageal malignancies. Once diagnosed, patients are nervous, anxious, and even desperate, which affects their sleep and mood, and the unfamiliar environment of the operating room aggravates this mood. In addition, this emotion causes preoperative sympathetic excitement. Owing to the retrospective nature of this study, preoperative anxiety level scores of patients could not be collected. The blood pressure measured at the time of admission was patients’ blood pressure when they were calm; most of them were nervous and anxious when entering the operating room, and the sympathetic tone decreased when sedative, analgesic, or intravenous anesthetic drugs were given. The difference between MAP at admission and that before the operation can indicate the patient’s preoperative mental tension and anxiety. A higher MAP difference indicates higher preoperative nervousness. In the present study, excessive preoperative stress and anxiety reduced sympathetic tone in patients after anesthesia induction, resulting in increased intraoperative urine volume. Increased sympathetic tone causes constriction of the renal arteries and decreased perfusion, consequently leading to decreased urine output. This suggests that a certain amount of preoperative psychological support and preoperative sedation are essential. In terms of the operation type, fluid loss is lesser in endoscopic surgery than in open surgery. Open surgery has more evaporation and blood loss, whereas endoscopic surgery allows retention of adequate blood volume and more urine. These findings may explain the higher likelihood of intraoperative polyuria in patients undergoing endoscopic surgery. Regarding the total intraoperative fluid intake, higher volumes of fluid infusion translate into higher urine volume. Greater blood loss during the operation is associated with a higher risk of a hemorrhagic shock, and the redistribution of body fluids may lead to a decreased urine output. The gradual increase in AVP concentration is reportedly related to the amount of blood loss but not to the rate of blood loss [32]. The increase in AVP concentration may be caused by changes in left atrial pressure, which decreases and causes a small decrease in blood volume; conversely, AVP concentration increases in response to the small decrease in blood volume [32]. The mechanism may be caused by the small decrease in blood volume stimulating an increase in renin secretion, followed by stimulation of AVP release by angiotensin II production [32]. The sensitivity of AVP and the renin–angiotensin system to volume stimulation suggests that volume stimulation is instrumental in the normal physiological control of these two hormonal systems [32]. Johnson et al. reported that elevated left atrial pressure reduces the release of plasma ADH, resulting in significantly increased urine volume [33]. This corroborates our finding that lesser intraoperative blood loss is associated with a higher probability of intraoperative polyuria. The use of vasopressor drugs will cause renal artery contraction and decrease renal blood flow, whereas without the use of vasopressor drugs, renal blood flow and urine volume increase. The nomogram clearly shows that the probability of intraoperative polyuria increases with decreases in cisatracurium use. To our knowledge, there is currently no study on cisatracurium causing intraoperative polyuria, and thus, further research is needed in this regard.

expandIn previous studies, intraoperative polyuria caused by dexmedetomidine use has been reported in many cases; however, in our trial, dexmedetomidine use did not influence the occurrence of intraoperative polyuria during thoracic general anesthesia surgery. Dexmedetomidine is an α-agonist that blocks the release of AVP and theoretically causes intraoperative polyuria [34]. The reasons for this may be the following. First, dexmedetomidine often significantly increases the heart rate, and thus, we use it in small doses and stop the infusion immediately when the patient develops sinus bradycardia; therefore, the dose of dexmedetomidine used intraoperatively is perhaps too small to cause intraoperative polyuria. Second, dexmedetomidine was administered by intraoperative pumping, and we could record an exact dose; therefore, a dichotomous variable was used to represent it. If we could record an exact dose as a continuous variable in the follow-up study, the results may have been different. In our study, all patients undergoing general anesthesia were continuously pumped with remifentanil and given sevoflurane via the inhalational route, and the total amount could not be recorded accurately but only as a dichotomous variable, and this may have influenced the results. Ketamine and propofol were also found to cause intraoperative polyuria in some patients in this study and were recorded as continuous variables, but no meaningful results were obtained in this regard. We also speculate the presence of drug–drug interactions, which remain to be explored in further clinical and basic studies. To our knowledge, this is the first time that a nomogram has been drawn for intraoperative polyuria in thoracic general anesthesia to easily calculate the risk for different individuals.

expandOur study has some limitations. First, since it was a retrospective study, many data were missing or unavailable, resulting in the exclusion of many samples. Second, we could not include more samples and more operation styles due to time and manpower constraints. Therefore, our model has limited potential for generalization and extrapolation. Third, there was some bias in defining and selecting candidate variables. Fourth, dexmedetomidine and vasopressors are administered using a micropump, which does not allow for recording their detailed doses, and therefore, unlike some drugs like fentanyl, they can only be recorded as dichotomous variables and not as a continuous variable; this may have an impact on the results. This suggests that our future clinical work needs to be more detailed and rigorous. Finally, the nature of retrospective studies may introduce bias and limit the establishment of causality. In the future, we need prospective studies as external validation to provide stronger evidence. However, taken together, although the nomogram has some limitations, it also can conveniently be a useful tool for predicting the probability of intraoperative polyuria in patients undergoing thoracic surgery. Owing to the traumatic nature of thoracic surgery, when analgesia is inadequate, the stress response is often reduced by additional fentanyl dosage. This retrospective study showed that the increase in fentanyl dosage increased the intraoperative urine output in patients. Owing to the widespread development of ultrasound-guided nerve block, thoracic paravertebral block and erector spinae block play an important role in analgesia for thoracic surgery. Findings of this study suggest that adequate preoperative nerve block can reduce the required dosage of intraoperative analgesic fentanyl and further reduce the occurrence of intraoperative polyuria. The application of preoperative sedative drugs can reduce the patient’s nervousness and anxiety and help reduce the incidence of intraoperative polyuria. Therefore, the use of preoperative sedative drugs is necessary. A restrictive infusion strategy also helps reduce the incidence of intraoperative polyuria. These are some of the avenues suggested by this trial for anesthesia management.

## Conclusions

Fentanyl, gender, the difference between MAP at admission and before the operation, operation type, total amount of fluids and blood products transfused, blood loss, vasopressor, and cisatracurium use were identified as predictors of intraoperative polyuria and incorporated into the nomogram. The nomogram shows good discrimination ability on the ROC curve and is well calibrated using the HL test. Decision curve analysis findings upheld the clinical usefulness of the nomogram. Individualized and precise prediction of intraoperative polyuria allows for better anesthesia management and early prevention optimization.

## Data Availability

No datasets were generated or analysed during the current study.

## Abbreviations

AIC: Akaike’s Information Criterion
ROC: Receiver Operating Characteristic
HL: Hosmer-Lemeshow Test
DCA: Decision Curve Analysis
HBP: Hypertension
CHD: Coronary Heart Disease
DM: Diabetes Mellitus
Cr: Creatinine
PLT: Platelet
Hb: Hemoglobin
MAP: Mean Arterial Pressure
HR: Heart Rate
IQR: Inter-Quartile Range
OR: Odds Ratio
CI: Confidence Interval
AVP: Arginine Vasopressin
VP: Vasopressin
ADH: Antidiuretic Hormone
GH: Growth Hormone

## References

[1] Wijnberge M, Geerts BF, Hol L, Lemmers N, Mulder MP, Berge P (2020). Effect of a machine learning-derived early warning system for Intraoperative Hypotension vs Standard Care on depth and duration of intraoperative hypotension during elective noncardiac surgery: the HYPE randomized clinical trial. JAMA.

[2] Van Decar LM, Reynolds EG, Sharpe EE, Harbell MW, Kosiorek HE, Kraus MB (2022). Perioperative Diabetes Insipidus caused by anesthetic medications: a review of the literature. Anesth Analg.

[3] Takeyama E, Ito C, Amano E, Shibuya H (2020). Increase in intraoperative urine output during tympanoplasty: a retrospective cohort study. Asian J Anesthesiol.

[4] Nigro N, Grossmann M, Chiang C, Inder WJ (2018). Polyuria-polydipsia syndrome: a diagnostic challenge. Intern Med J.

[5] Monaghan TF, Rahman SN, Bliwise DL, Michelson KP, Agudelo CW, Miller CD (2020). Identifying men with global polyuria on a nocturnal-only voiding diary. Neurourol Urodyn.

[6] D’Ancona C, Haylen B, Oelke M, Abranches-Monteiro L, Arnold E, Goldman H (2019). The International Continence Society (ICS) report on the terminology for adult male lower urinary tract and pelvic floor symptoms and dysfunction. Neurourol Urodyn.

[7] Adams PS, Cassara A (2016). Dexmedetomidine-related polyuria in a pediatric patient. J Anesth.

[8] Uddin MM, Sebastian J, Usama M, Raziq FI, Saydain G, Rossi NF (2021). Dexmedetomidine Induced Polyuria in the Intensive Care Unit. Case Rep Crit Care.

[9] Vani S, Stackpole A, Kovacevic MP. Probable Dexmedetomidine Induced Diabetes Insipidus: a Case Review. J Pharm Pract. 2021:8971900211053261. 10.1177/08971900211053261 34670426

[10] Villela NR, do Nascimento Júnior P, de Carvalho LR, Teixeira A (2005). Effects of dexmedetomidine on renal system and on vasopressin plasma levels. Experimental study in dogs. Rev Bras Anestesiol.

[11] Greening A, Mathews L, Blair J (2011). Apparent dexmedetomidine-induced polyuric syndrome in an achondroplastic patient undergoing posterior spinal fusion. Anesth Analg.

[12] Pratt A, Aboudara M, Lung L (2013). Case report: polyuria related to dexmedetomidine. Anesth Analg.

[13] Ji F, Liu H (2013). Intraoperative hypernatremia and polyuric syndrome induced by dexmedetomidine. J Anesth.

[14] Xu A, Wan L (2018). Dexmedetomidine-induced polyuric syndrome and hypotension. J Clin Anesth.

[15] Granger S, Ninan D (2017). Intraoperative Dexmedetomidine-Induced Polyuric Syndrome. Cureus.

[16] Hatab SZ, Singh A, Felner EI, Kamat P (2014). Transient central diabetes insipidus induced by ketamine infusion. Ann Pharmacother.

[17] Gaffar S, Eskander JP, Beakley BD, McClure BP, Amenta P, Pierre N (2017). A case of central diabetes insipidus after ketamine infusion during an external to internal carotid artery bypass. J Clin Anesth.

[18] Kataria V, Kang T, Bradley KM (2018). Ketamine-Induced Diabetes Insipidus. J Pain Palliat Care Pharmacother.

[19] Herity LB, Baker C, Kim C, Lowe DK, Cahoon WD (2021). Jr. Delayed onset of Central Diabetes Insipidus with ketamine sedation: a report of 2 cases. J Pharm Pract.

[20] Kassebaum N, Hairr J, Goldsmith W, Barwise J, Pandharipande P (2008). Diabetes insipidus associated with propofol anesthesia. J Clin Anesth.

[21] Soo J, Gray J, Manecke G (2014). Propofol and diabetes insipidus. J Clin Anesth.

[22] Hong JC, Ramos E, Copeland CC, Ziv K. Transient intraoperative Central Diabetes Insipidus in Moyamoya patients undergoing revascularization surgery: a Mere coincidence? A A Case Rep. 2016;6:224–7. 10.1213/XAA.0000000000000287 26795912

[23] Inoue Y, Shibuya I, Kabashima N, Noguchi J, Harayama N, Ueta Y (1999). The mechanism of inhibitory actions of propofol on rat supraoptic neurons. Anesthesiology.

[24] Zhou ZB, Yang XY, Yuan BL, Niu LJ, Zhou X, Huang WQ (2015). Sevoflurane-induced down-regulation of hippocampal oxytocin and arginine vasopressin impairs juvenile social behavioral abilities. J Mol Neurosci.

[25] Shimogai M, Ogawa K, Tokinaga Y, Yamazaki A, Hatano Y (2010). The cellular mechanisms underlying the inhibitory effects of isoflurane and sevoflurane on arginine vasopressin-induced vasoconstriction. J Anesth.

[26] Ohara S, Nishimura A, Tachikawa S, Iijima T (2020). Effect of remifentanil on intraoperative fluid balance: a retrospective statistical examination of factors contributing to fluid balance. J Dent Anesth Pain Med.

[27] Lehtinen AM, Fyhrquist F, Kivalo I (1984). The effect of fentanyl on arginine vasopressin and cortisol secretion during anesthesia. Anesth Analg.

[28] Kim SY, Yoon MJ, Park YI, Kim MJ, Nam BH, Park SR (2018). Nomograms predicting survival of patients with unresectable or metastatic gastric cancer who receive combination cytotoxic chemotherapy as first-line treatment. Gastric Cancer.

[29] de Lange S, Boscoe MJ, Stanley TH, de Bruijin N, Philbin DM, Coggins CH (1982). Antidiuretic and growth hormone responses during coronary artery surgery with sufentanil-oxygen and alfentanil-oxygen anesthesia in man. Anesth Analg.

[30] Stanley TH, Philbin DM, Coggins CH (1979). Fentanyl-oxygen anaesthesia for coronary artery surgery: cardiovascular and antidiuretic hormone responses. Can Anaesth Soc J.

[31] Kono K, Philbin DM, Coggins CH, Moss J, Rosow CE, Schneider RC (1981). Renal function and stress response during halothane or fentanyl anesthesia. Anesth Analg.

[32] Claybaugh JR, Share L (1973). Vasopressin, renin, and cardiovascular responses to continuous slow hemorrhage. Am J Physiol.

[33] Johnson JA, Moore WW, Segar WE (1969). Small changes in left atrial pressure and plasma antidiuretic hormone titers in dogs. Am J Physiol.

[34] Shirasaka T, Kannan H, Takasaki M (2007). Activation of a G protein-coupled inwardly rectifying K + current and suppression of Ih contribute to dexmedetomidine-induced inhibition of rat hypothalamic paraventricular nucleus neurons. Anesthesiology.

